# A Novel Intumescent MCA-Modified Sodium Silicate/Acrylic Flame-Retardant Coating to Improve the Flame Retardancy of Wood

**DOI:** 10.3390/molecules29133021

**Published:** 2024-06-26

**Authors:** Yuting Wang, Ying Ran, Yuran Shao, Jiawei Zhu, Chungui Du, Fei Yang, Qichao Bao, Yingying Shan, Weigang Zhang

**Affiliations:** Bamboo Industry Institute, Zhejiang A & F University, Hangzhou 311300, China; wang270229@163.com (Y.W.); ry18119295807@163.com (Y.R.); shao18309819091@163.com (Y.S.); yangfeier0826@163.com (F.Y.); bqc1125573308@gmail.com (Q.B.); syy15968566686@163.com (Y.S.); 20091018@zafu.edu.cn (W.Z.)

**Keywords:** waterborne acrylic acid, flame-retardant coating, sodium silicate, melamine cyanurate (MCA), flame-retardant mechanism

## Abstract

The incompatibility between inorganic flame retardants and organic acrylic coatings represents a significant challenge that requires resolution. This work selected environmentally friendly organic aqueous acrylic coatings as the substrate, sodium silicate hydrate as the inorganic flame retardant, and melamine cyanurate (MCA) as the flame-retardant modifier and the flame-retardant co-modifier, with the objective of improving the dispersion and flame-retardant properties of sodium silicate hydrate in the aqueous acrylic coatings. Subsequently, the sodium silicate/MCA/waterborne acrylic acid flame-retardant coating was prepared. The flame-retardant treatment was then applied to poplar veneer in order to create a flame-retardant poplar veneer. The dispersion of the flame-retardant coating was characterized by scanning electron microscopy (SEM), energy-dispersive spectroscopy (EDX), and X-ray diffractometry (XRD). Furthermore, the flame-retardant properties of the flame-retardant poplar veneer were analyzed by thermogravimetry (TG), limiting oxygen index (LOI), and cone calorimeter. The results demonstrated that the MCA-modified sodium silicate flame retardant was well dispersed in aqueous acrylic coatings. The results of the flame-retardant properties of the poplar veneer indicated that the ignition time of the 9% flame retardant-treated poplar veneer was increased by 122.7%, the limiting oxygen index value was increased by 43.0%, and the peak heat release rate (pHRR), the peak total heat release rate (pTHR), and the peak mass loss rate were decreased by 19.9%, 10.8%, and 27.2%, respectively, in comparison to the non-flame retardant-treated poplar veneer. Furthermore, the residual char mass increased by 14.4%, and the residual char exhibited enhanced thickness, density, and regularity. The results demonstrated that MCA was an effective promoter of sodium silicate dispersion in acrylic coatings. Furthermore, the sodium silicate/MCA/waterborne acrylic flame-retardant coating significantly enhance the flame retardancy of wood, and its flame retardant mechanism was consistent with the synergistic silicone–nitrogen expansion flame-retardant mechanism. This work presents a novel approach to enhancing the dispersion of inorganic flame retardants in organic coatings, offering a valuable contribution to the advancement of research and application in the domains of innovative flame retardant coatings and flame retardant wood.

## 1. Introduction

Wood and its products have long been used as both decorative and building material due to their low density, low cost, and good mechanical performance. However, the inherent flammability of wood increases its fire hazard, resulting in a large number of casualties and performance losses. This has led to the extremely restricted application of wood in some fields, particularly in densely populated areas [1,2]. It is therefore evident that the materials in question are of paramount importance in addressing the fire safety requirements. In the case of existing timber structures are involved, the most usual way to improve its reaction to fire is to treat wood with flame-retardant materials [3]. Numerous strategies have been employed, including soaking with flame retardants and applying flame-retardant coatings to the wood surface. These have been utilized to improve the fire protection performance of wood [4]. In particular, the method of applying flame-retardant coatings to wood surfaces has considerable potential, offering the advantages of a simple process, low cost, and ease of mass production [5,6,7].

Flame-retardant coatings are currently divided into organic and inorganic variants, according to the base material. Additionally, the composition and nature of the flame-retardant coating can be further subdivided into oil and waterborne flame-retardant coatings [8,9,10]. Waterborne coatings are a type of environmentally friendly coating that uses water as the main solvent [11,12]. In comparison to traditional solvent-based wood coatings, waterborne coatings offer several advantages, including non-toxicity and environmental protection, and the absence of a smell, minimal volatile matter, non-combustibility and explosion, and the prevention of yellowing and large painting areas [13,14,15]. These advantages have led to an increasing market acceptance of waterborne coatings. However, despite their environmental benefits, waterborne coatings have yet to be widely adopted in the paper packaging industry for food and pharmaceutical products. To date, there has been a paucity of systematic studies on the application of waterborne coatings to wood materials. Further research is therefore required to address this gap in knowledge.

The most commonly used method for preparing flame-retardant coatings is to add inorganic flame retardants to the coating. There are numerous types of wood material flame retardants, which can be divided into inorganic and organic flame retardants according to their chemical composition [16,17]. Inorganic flame retardants are advantageous in terms of their excellent flame-retardant performance, simple technology, and low cost. They have been extensively studied and widely applied. Sodium silicate, also known as water glass, is an example of an inorganic flame retardant [18]. It is favored by academia and industry due to its advantages of good environmental performance, high flame-retardant performance, and low cost [19,20]. Sodium silicate functions as a flame retardant, primarily by forming an insulating layer of inorganic silica residue on the surface of the wood during combustion. This is achieved through the hydrolysis of sodium silicate, which forms polysilicic acid and an inorganic film. This film acts as a barrier layer on the surface of the wooden material, preventing the escape of combustible gases and the entry of oxygen into the wood. However, crystallization is the most common method for the formation of inorganic materials. As an inorganic material, sodium silicate always produces nano- or micro-sized solid powders rather than bulks with continuous structure [21]. In contrast, waterborne acrylic coating is an organic material and has an easy and moldable fabrication process, which can generate large-sized bulk materials with continuous structures [22]. Inorganic materials are easily crystallized in organic polymers, and they are difficult to disperse well due to the totally distinct formation pathways of inorganic and organic components. Consequently, sodium silicate is unable to form a uniform heat insulation layer, which results in poor flame-retardant performance, when it is added into waterborne acrylic coating. The precise and uniform incorporation of sodium silicate into waterborne acrylic coating remains a significant challenge at this time [23]. Melamine cyanurate (MCA) contains a significant number of amino groups, and the hydrogen bond formed between the amino groups of MCA and sodium silicate can effectively hinder the polymerization of sodium silicate. Furthermore, MCA is an environmentally friendly nitrogen-based flame retardant that can be used as a blowing agent and lubricant. The incorporation of low quantities of MCA into a polymer can enhance the flame-retardant properties of the polymer, while maintaining the mechanical properties of the polymer. Consequently, MCA acts as a modifier and co-flame retardant for sodium silicate, thereby improving the polymerization phenomenon of sodium silicate and enhancing its flame retardancy.

The objective of this study was to improve the dispersion and flame retardant properties of sodium silicate hydrate in aqueous acrylic coatings by incorporating melamine cyanurate (MCA) as a flame-retardant modifier and co-modifier. The environmentally friendly organic substrate was chosen for its potential to enhance the flame-retardant properties of sodium silicate hydrate. The sodium silicate/MCA/aqueous acrylic flame-retardant coating was subjected to analysis by scanning electron microscopy (SEM) and Raman, in order to ascertain whether the compatibility between the inorganic flame retardant and the organic coating had been enhanced. Subsequently, the effects of the hydrated sodium silicate/MCA/waterborne acrylic flame-retardant coatings on the flame-retardant properties of poplar veneer were evaluated in a systematic manner through the use of the limiting oxygen index (LOI) test and cone calorimeter test. Furthermore, the dispersion mechanism and flame-retardant mechanism of the novel hydrated sodium silicate/MCA/waterborne acrylic flame retardant coating were also analyzed. This study provides theoretical guidance for the development of high-performance flame-retardant water-based coatings and is of great significance for the comprehensive promotion flame-retardant coating applications in the fields of wood, furniture, and architecture.

## 2. Results and Discussion

### 2.1. Appearance of Sodium Silicate/MCA/Waterborne Acrylic Flame-Retardant Coating

**Figure 1:**
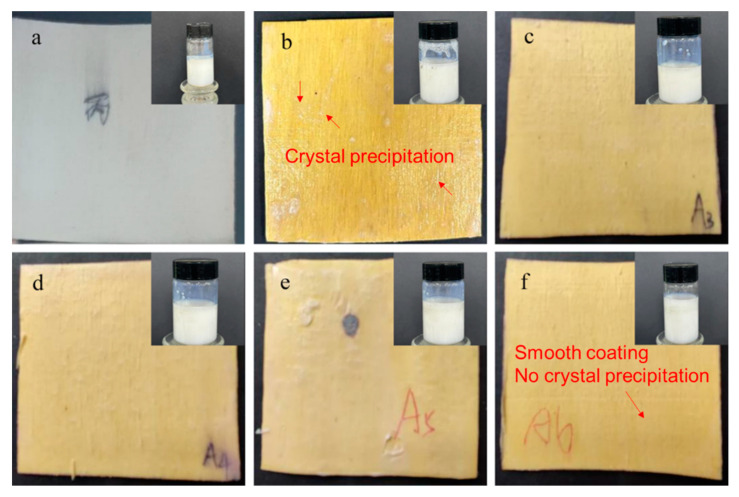
Appearance of waterborne acrylic flame-retardant coating and its flame-retardant poplar veneer. (**a**–**f**) Poplar veneer treated with MP-0, MP-1, MP-2, MP-3, MP-4, and MP-5, respectively.

Figure 1 illustrates the appearance morphology of six waterborne acrylic flame-retardant coatings (MP-0, MP-1, MP-2, MP-3, MP-4, and MP-5) prepared and flame-retardant poplar veneers. It can be observed that the color of the waterborne acrylic coating and the waterborne acrylic flame-retardant coating produced by the addition of different proportions of flame retardant is milky white. Consequently, the incorporation of the flame retardant does not alter the original color of the waterborne acrylic coating. The waterborne acrylic flame retardant coating does not exhibit any signs of crystallization, agglomeration, or other such phenomena; rather, it is uniformly dispersed in a lotion-like form. Figure 1 illustrates that the incorporation of MCA into sodium silicate/acrylic coatings markedly enhances the dispersion of sodium silicate within acrylic coatings, thereby resolving the issues of facile crystallization and agglomeration of sodium silicate in aqueous acrylics. The addition of MCA to sodium silicate results in the formation of hydrogen bonds between the amine group in MCA and sodium silicate, which hinders the polymerization of sodium silicate. Furthermore, as illustrated in Figure 1, the poplar veneer coated with waterborne acrylic coating appears white, which is consistent with the color of the waterborne acrylic coating. The color of the poplar veneer coated with sodium silicate and waterborne acrylic acid appears dark yellow. In contrast, the poplar veneer coated with waterborne acrylic flame retardant coating appears yellow. However, the color of the latter is lighter than that of the former, which is consistent with the lighter color of MPW-1.

### 2.2. Viscosity and Film Adhesion of Sodium Silicate/MCA/Waterborne Acrylic Flame-Retardant Coating

**Figure 2:**
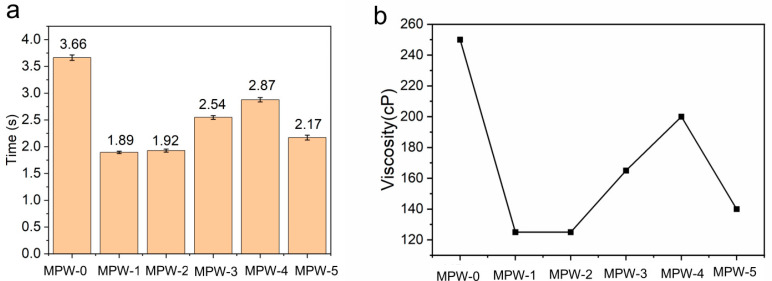
(**a**) Format tube viscometer measurement time; (**b**) viscosity values of sodium silicate/MCA/waterborne acrylic flame-retardant coating.

The viscosity of the different coatings was measured using a format tube viscometer, with the following results: 3.67 s for MP-0, 1.89 s for MP-1, and 1.92 s, 2.54 s, 2.87 s, and 2.17 s for MP-2 to MP-5, respectively. The viscosity change obtained from the viscosity conversion table is shown in Figure 2a. The results of viscosity measurements of different sodium silicate/MCA/waterborne acrylic flame-retardant coatings using a format tube viscometer are shown in Figure 2b. It can be seen that the addition of sodium silicate nine-hydrate reduces the viscosity of waterborne acrylic coating, which is because sodium silicate can form hydrogen bonds with water molecules, thereby increasing the dispersibility of waterborne coating and reducing viscosity [24]. When MCA is added, the hydrogen bonding between the amino group of MCA and sodium silicate hinders the polymerization of sodium silicate, and the hydrogen bonding reaction weakens the effect of sodium silicate on reducing viscosity. Therefore, the viscosity of the coating begins to show an upward trend. However, when the hydrogen bonding reaction reaches saturation, continuing to increase MCA will no longer increase the viscosity of the coating, so excess sodium silicate will continue to form hydrogen bonds with water molecules, reducing viscosity. It can be observed that the viscosity of waterborne acrylic flame-retardant coating varies with different flame retardant ratios. As the flame retardant ratio increases from 5% to 9%, the viscosity of waterborne acrylic flame-retardant coating gradually increases. Conversely, when the flame retardant ratio increases from 9% to 11%, the viscosity of waterborne acrylic flame-retardant coating begins to decrease. When the flame-retardant ratio is 9% (MP-4), the viscosity value of the flame-retardant coating is comparable to that of the coating without flame retardant (MP-0). This suggests that sodium silicate nine-hydrate can reduce the viscosity of waterborne acrylic coating, while the addition of MCA has the opposite effect, indicating a potential interaction between MCA and the system.

The hardness of the paint film on poplar veneer under different coatings and the adhesion of each coating are measured using a pencil hardness tester, as shown in Table 1. The hardness of the paint film is an important performance indicator of the mechanical strength of coatings, and it is also an important item to measure the quality of paint products [25]. It can be demonstrated that the hardness of a paint film is directly proportional to its wear resistance, compressive strength, and chemical resistance [26]. In the hardness test, coatings are graded based on the number of pencil scratches or the damage caused by various hardness levels. The grading gradually decreases from the hardest 6H through 5H, 4H, 3H, 2H, and H, then passes through the moderately soft and hard HB, and then moves from B and 2B to the softest 6B. Table 1 illustrates that MPW-0 is HB, indicating that the coating has moderate softness and hardness. After the addition of sodium silicate nine-hydrate, the paint film hardness of MPW-1 remained almost unchanged, reaching the HB level. After the addition of MCA, the hardness of the paint film decreased slightly, and MPW-2 reached the H level. However, as the quantity of MCA is augmented, the hardness of the paint film also increases to the HB level. This indicates that the addition of flame retardant has a negligible effect on the paint film hardness of flame-retardant poplar veneer, and its paint film hardness is approximately HB.

The adhesion of paint film refers to the ability of the paint film to bond with the surface of the coated object or between coatings. Adhesion is a crucial technical indicator and a fundamental prerequisite for paint films to exhibit a range of performance characteristics. A paint film with good adhesion is more durable. Conversely, paint films with poor adhesion are prone to cracking and peeling, rendering them unusable. “Determination of Adhesion of Paint Films”, a paint film is deemed to have met the requisite ASTM grade of 4B if, during the adhesion test, small pieces of peeling are observed at the intersection of the cuts on the coating, and the actual damage within the grid area does not exceed 5% [27]. Table 1 indicates that the paint film adhesion of the six coatings can be classified as 4B, thereby indicating that the paint film adhesion test is qualified.

### 2.3. Ignition Testing of Flame-Retardant Veneer

**Figure 3:**
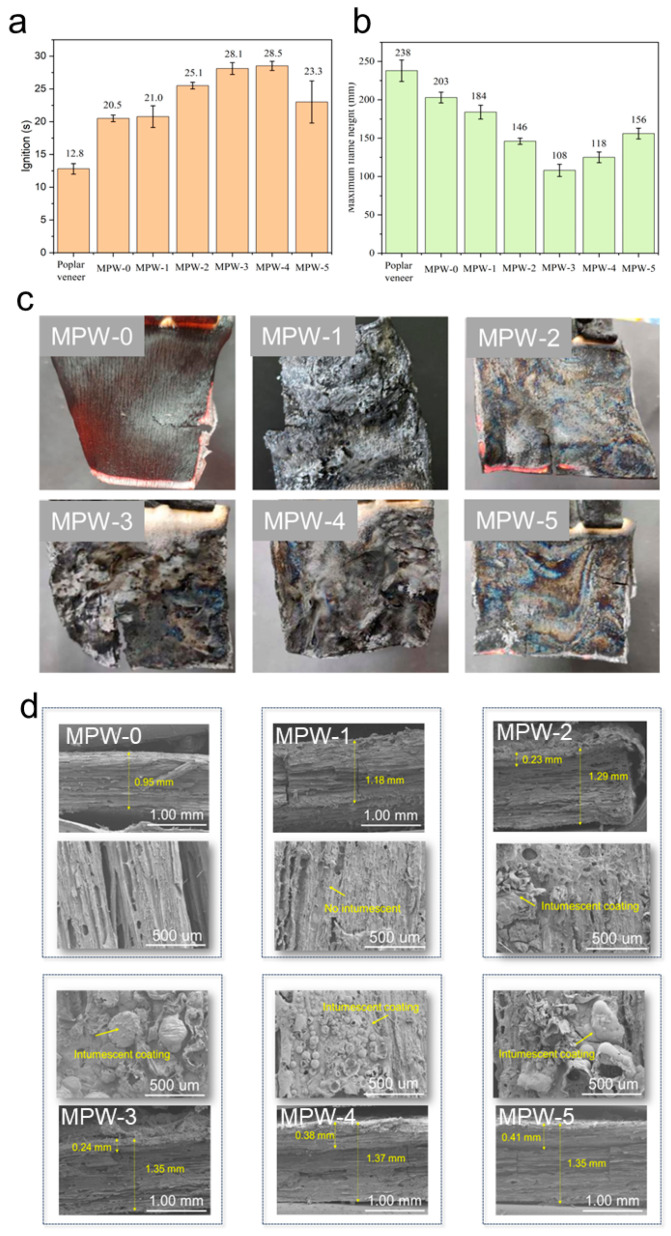
(**a**) The ignition time of poplar veneer before and after flame-retardant treatment; (**b**) the maximum flame height of poplar veneer before and after flame-retardant ignition. (**c**) Residual carbon of poplar veneer treated with MPW-0, MPW-1, MPW-2, MPW-3, MPW-4, and MPW-5, respectively. (**d**) SEM surface and cross-section of residual carbon in poplar veneer treated with MPW-0, MPW-0, MPW-1, MPW-2, MPW-3, MPW-4, and MPW-5.

The ignition time and maximum flame height of the flame-retardant veneer are shown in Figure 3a. It can be seen that the ignition time of poplar veneer is 12.8 s when it has no coating protection. The ignition time of MPW-0 is 20.5 s, which has a certain flame-retardant effect, but it is worse than that of the flame-retardant poplar veneers. The ignition time of MPW-1 is increased by 0.5 s in comparison to MPW-0. As the flame retardant ratio is increased from 5% to 9%, the ignition time of the flame-retardant poplar veneer gradually increases, and the flame-retardant effect improves. The ignition time of the flame-retardant poplar veneer (MPW-4) coated with a flame retardant ratio of 9% is 28.5 s, which is extended by 15.7 s and 122.7%, demonstrating a satisfactory flame-retardant effect. Conversely, when increasing from 9% to 11%, the ignition time of flame-retardant poplar veneer decreases, and the flame-retardant effect deteriorates. This suggests that sodium silicate nine-hydrate can enhance the flame retardancy of poplar veneer to a certain extent. However, the incorporation of MCA markedly enhances the flame retardancy of the composite flame-retardant system comprising sodium silicate nine-hydrate and MCA. Furthermore, the flame retardant ratio of 9% exhibits the most effective flame-retardant properties.

Figure 3b demonstrates that the maximum flame height of poplar veneer during ignition testing is 238 mm. The maximum flame height of poplar veneer coated with waterborne acrylic acid during ignition testing was 203 mm, while the maximum flame height of MPW-1 during ignition testing was 184 mm. The maximum flame height of Class B1 flame-retardant materials is less than or equal to 150 mm [28]. This indicates that poplar veneer, MPW-0, and MPW-1 lack flame-retardant performance and are therefore combustible materials. Furthermore, the maximum flame heights of MPW-2, MPW-3, and MPW-4 are 146 mm, 108 mm, and 125 mm, respectively. These values are below the B1 level requirements of the national standard for fire-resistant materials and correspond to the category of fire-resistant materials. It can be demonstrated that the incorporation of MCA has a significant impact on the flame-retardant performance of the flame-retardant coating, which is in alignment with the findings of the ignition time (Figure 2). Consequently, the flame-retardant poplar veneer under investigation in this study exhibits remarkable flame-retardant capabilities.

The residual carbon and SEM of the fire-retardant poplar veneer after ignition test are shown in Figure 3c and Figure 3d, respectively. As illustrated in Figure 3, the residual mass of MPW-0 following combustion is relatively minimal, with only a thin layer of residual carbon remaining in a curved shape, exhibiting cracks in the middle. The residual mass of MPW-1 and the thickness of the residual carbon layer are both greater than MPW-0 (0.95 mm), indicating that the addition of flame-retardant sodium silicate increases the mass of residual carbon, thereby improving the flame-retardant performance of poplar veneer. The flame-retardant poplar veneers with a flame retardant ratio of 5% (MPW-2) and 11% (MPW-5) exhibited a complete residual carbon layer after combustion, which essentially maintained the original morphology of the poplar veneer. The thickness of the residual carbon was greater than that of MPW-0, indicating that the flame-retardant poplar veneer exhibited excellent flame-retardant performance. The flame-retardant poplar veneer with the flame retardant ratio of 7% (MPW-3) and 9% (MPW-4) has a significantly expanded carbon layer, and the thickness of the carbon residue layer increased significantly after combustion, which is probably because of the presence of a large amount of nitrogen in melamine cyanurate (MCA) [29]. The flame retardant produces hard-to-burn carbon foam when it chemically interacts with flammable gas. When this carbon foam is exposed to the flame, it quickly absorbs heat and forms a dense coating that has a certain strength. Because the poplar veneer is coated with a flame retardant, it can prevent the gas products generated by decomposition from escaping from the surface of the material, causing the system, which is already in a molten state, to expand and foam, thus forming a thicker expanded carbon layer, greatly improving the flame-retardant performance of the poplar veneer. As can be seen in Figure 3d, a dense carbon layer was formed after the combustion of the flame-retardant poplar veneer, which was attributed to the formation of uniform SiO_2_ on the surface of the wood after the combustion of sodium silicate [30]. In summary, the poplar veneer treated with waterborne acrylic flame-retardant coating has good flame-retardant performance, and the optimum ratio of flame retardant in waterborne acrylic flame-retardant coating is 9%.

### 2.4. Limiting Oxygen Index (LOI) Analysis

The LOI is an “ease of extinction” test performed in a flowing oxygen/nitrogen mixture and measures the minimum oxygen concentration required to support downward flame propagation [31]. The limiting oxygen index test results of poplar veneer before and after flame-retardant treatment are shown in Table 2. A comparison of the limiting oxygen indices of the various materials reveals that MPW-0 has the lowest value (20.0%), while MPW-4 has the highest (28.6%), representing a 43% increase over that of MPW-0. The limiting oxygen index of MPW-1 is 27.0%, which is 35.0% higher than MPW-0, but all are lower than the limiting oxygen index values of composite flame-retardant coatings (MPW-2, MPW-3, and MPW-4) without MCA application. The results of this study indicate that an increase in the ratio of flame retardant from 5% to 9% in a waterborne acrylic flame-retardant coating has the effect of increasing the limiting oxygen index of flame-retardant poplar veneer and enhancing flame-retardant performance. Conversely, an increase in the ratio of flame retardant to 11% has the effect of reducing the limiting oxygen index of flame-retardant poplar veneer, accompanied by a deterioration in flame-retardant performance. This is consistent with the observed change in ignition time (Figure 2). The results demonstrate that the flame retardant, prepared by modifying sodium silicate with MCA, significantly enhances the flame-retardant performance of waterborne acrylic coatings. However, when the ratio of flame retardant reaches a specific threshold, its flame-retardant performance can actually decline. One possible explanation for this observation is that the excessive amount of flame retardant added impairs its uniform distribution on poplar veneers. Consequently, when the ratio of MCA-modified sodium silicate flame retardant in waterborne acrylic flame-retardant coating reaches 5%, 7%, and 9%, the flame-retardant poplar veneer produced is deemed to meet the requirements of flame-retardant materials [32].

### 2.5. Characterization of Flame-Retardant Coatings

**Figure 4:**
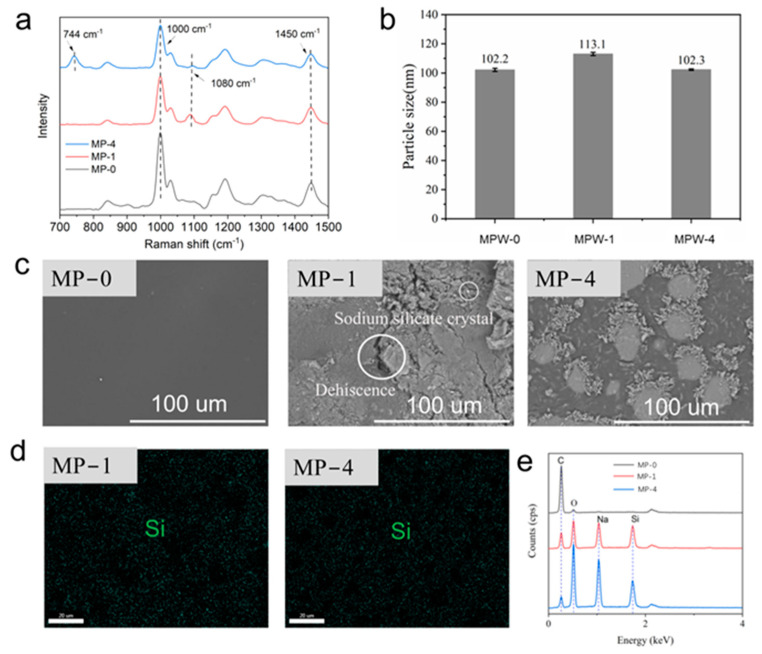
(**a**) XRD diagram of sodium silicate nine-hydrate and different proportions of flame-retardant aqueous solutions. (**b**) Raman spectra of MPW-0, MPW-1, and MPW-4 coatings; (**c**) particle sizes of MP-0, MP-1, and MP-4 materials; (**d**) SEM images of MP-0, MP-1, and MP-4 front; (**e**) Si element distribution map and EDX of MP-1 and MP-4 coatings.

The XRD spectra of sodium silicate, sodium silicate/acrylic coating, and sodium silicate/MCA/acrylic flame-retardant coating are shown in Figure 4a. As illustrated in Figure 4a, the 26.2° peak observed in the sodium silicate nine-hydrate flame-retardant coating prepared with sodium silicate/MCA flame retardant with different flame retardant ratios has disappeared. The XRD peak was no longer sharp, particularly in the group with a flame retardant ratio of 7%, which exhibited a “Mantou peak” [33]. This indicated that the addition of MCA significantly reduced the crystallinity of the flame retardant, resulting in more uniform dispersion of sodium silicate nine-hydrate. This is consistent with the analysis results of appearance morphology and SEM, which once again confirms that the addition of MCA can significantly improve the uniform dispersion performance of sodium silicate nine-hydrate flame retardant in waterborne acrylic coating.

According to the previous work, the MP-4 formulation can be prepared as a sodium silicate/MCA/waterborne acrylic flame-retardant coating with excellent flame-retardant and dispersion properties. Thus, the MP-0, MP-1, and MP-4 are further analyzed.

Raman spectra of MP-0, MP-1, and MP-4 coatings are shown in Figure 4b. The peak located at 1080 cm^−1^ corresponds to the Si-O stretching vibration, which is attributed to sodium silicate [34]. The Raman shift at 744 cm^−1^ corresponds to the characteristic peak of the cyanuric ammonia molecule [35]. The characteristic peak at 1000 cm^−1^ is attributed to the asymmetric telescopic vibration of C-C. The peak at 1450 cm^−1^ is assigned to -OH group bending vibrations because water is present in the acrylic acid [36]. This result indicates that the introduction of sodium silicate and MCA is without altering the chemical structure of acrylic acid. MP-0, MP-1, and MP-4 coatings were selected for particle size testing, and the results are shown in Figure 4c. It can be seen that the particle size of waterborne acrylic coating increases when sodium silicate nine-hydrate is added. The addition of MCA results in a reduction in the particle size of the coating. This indicates that the incorporation of MCA effectively inhibits the drying crystallization and aggregation of sodium silicate hexahydrate, thereby promoting the uniform dispersion of sodium silicate in waterborne acrylic flame-retardant coatings. This is consistent with the analysis results obtained from the examination of the coating’s appearance morphology, SEM, EDX, XRD, and other techniques.

Figure 4d displays SEM images of MP-0, MP-1, and MP-4. It can be observed that the surface of MPW-0 is smooth, and the coating is evenly dispersed. In contrast, the surface of MPW-1-treated poplar veneer exhibited cracking, which was attributed to the reduction in the film-forming property of acrylic acid resulting from the agglomeration of sodium silicate. The poplar veneer treated with a flame-retardant coating (MP-4) prepared by the addition of melamine cyanurate exhibited a uniform distribution of the coating on the surface and an absence of cracking. This indicates that the addition of MCA reacts with sodium silicate hydrate, alleviating the aggregation and cracking caused by sodium silicate hydrate aggregation. This phenomenon further corroborates the analysis of appearance morphology. The possible reason for this may be due to the easy polymerization of sodium silicate in waterborne acrylic coating, while the addition of MCA combines with sodium silicate to form a stable structure, reducing the crystallinity of sodium silicate and forming a uniform coating. The EDX surface scans of MP-1 and MP-4 are shown in Figure 4e, which shows that the distribution of Si elements in MP-4 is more homogeneous compared to that in MP-1. The silicate ions in sodium silicate nine-hydrate polymerize with MCA to form a SiO_2_ layer, which hinders the drying and crystallization of sodium silicate nine-hydrate and makes it more evenly dispersed in the coating after the combination of the two. The results of elemental analysis are consistent with those of SEM and morphology analysis, which once again confirms that the addition of MCA can significantly improve the uniform dispersion performance of sodium silicate nine-hydrate flame retardant in waterborne acrylic coating.

### 2.6. Flame-Retardant Properties of Flame-Retardant Poplar Veneer Analysis

**Figure 5:**
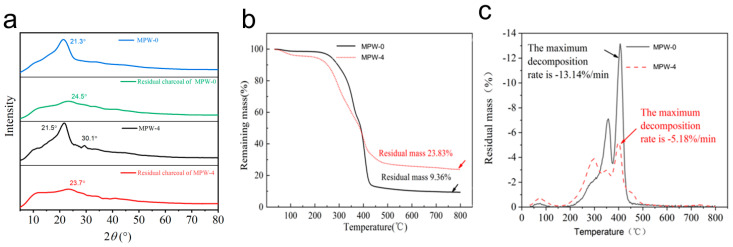
(**a**) XRD spectra of MPW-0 and MPW-4 before and after burning; (**b**)TG of MPW-0 and MPW-4; (**c**) DTG of MPW-0 and MPW-4.

The XRD spectra of poplar veneer treated with MP-0 and MP-4, respectively, before and after combustion, are presented in Figure 5a. The characteristic diffraction peaks at 21.3° and 21.5° were attributed to cellulose in the poplar veneer, which disappeared in the poplar veneer after combustion in comparison to the sample before combustion, suggesting pyrolysis of cellulose [37]. The emergence of novel low “bun” peaks at 24.5° and 23.7°, which are ascribed to the (101) crystal plane of sodium silicate, indicates that sodium silicate undergoes the formation of SiO_2_ at elevated temperatures. Furthermore, the distinctive diffraction peak at 30.1° observed in the MP-4-treated poplar veneer was attributed to MCA [38], which was absent following combustion due to the flame-retardant effect of MCA in conjunction with sodium silicate.

The fundamental principle of a thermogravimetric analyzer is to record the mass changes in measured substances across different temperature ranges and to obtain the decomposition rate of the substances in each stage of the decomposition process [39]. The derivative obtained by taking the first derivative of the thermogravimetric curve is the thermogravimetric rate curve (DTG) of the experimental sample during the experimental process [40].

As shown in the TG diagram in Figure 5a, the initial weight loss temperature of poplar veneer treated with MP-4 flame retardant was found to be lower than that of untreated poplar veneer. At the conclusion of the combustion process, the residual mass fraction of the flame retardant-treated poplar veneer was 23.8%, which was considerably higher than that of the untreated poplar veneer (9.4%), and the residual mass increased by 14.4%. As illustrated in Figure 5b, the mass loss rate of the flame retardant-treated poplar veneer is obviously lower than that of untreated poplar veneer. The maximum decomposition rate of the flame retardant-treated poplar veneer was 5.2%/min, which was significantly lower than that of the untreated poplar veneer, with a maximum decomposition rate that was 7.9%/min lower. Consequently, poplar veneer treated with a sodium silicate/MCA/waterborne acrylic flame-retardant coating exhibits excellent thermal stability.

In order to provide a more systematic evaluation of flame retardancy, cone calorimetry tests were conducted on poplar veneer treated with MP-0, MP-1, and MP-4. The mean parameter values of CONE for samples are shown in Table 3.

**Figure 6:**
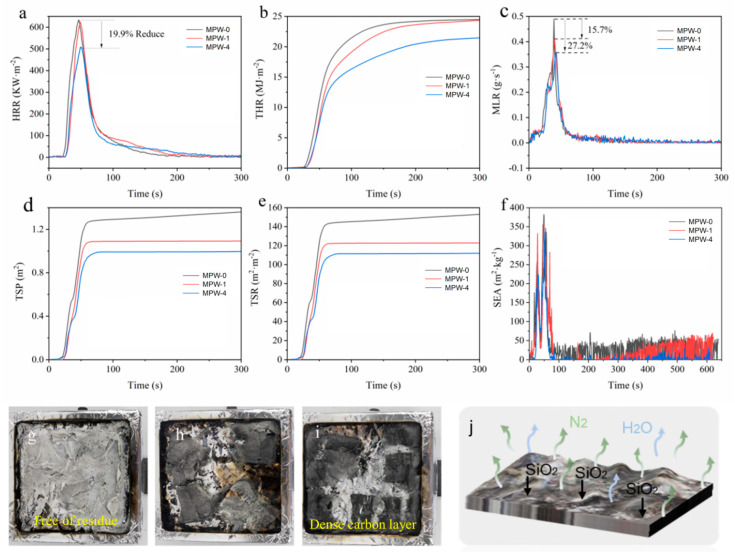
Cone calorimetry test results and digital photos of carbon residue of flame-retardant coatings. (**a**) HRR, (**b**) THR, (**c**) MLR, (**d**) TSP, (**e**) TSR, (**f**) SEA, (**g**) carbon residue of MPW-0, (**h**) carbon residue of MPW-1, (**i**) carbon residue of MPW-4, and (**j**) mechanism of flame-retardant action.

Heat release rate (HRR) refers to the amount of heat released per unit time during material combustion and is one of the most important parameters for determining the fire hazard of materials. Figure 6a illustrates that the exothermic peak of MPW-4 is significantly smaller than that of the two control groups (MPW-0 and MPW-1). The peak of the exothermic peak of MPW-4 (508 KW·m^−2^) is 19.9% lower than that of MPW-0 (634 KW·m^−2^). This indicates that the addition of MCA-modified sodium silicate flame retardant significantly improves the flame retardancy of waterborne acrylic coating, thereby effectively suppressing the combustion of poplar veneers.

Total heat release (THR) is a parameter that describes the total amount of heat released per unit area of a sample from the beginning to the end of combustion. When combined with the HRR, it can provide a more comprehensive evaluation of the combustion characteristics of materials. Figure 6b illustrates that the total heat release (THR) of MPW-4 and the two control groups (MPW-0 and MPW-1) increased with time throughout the entire combustion process. In comparison to MPW-0, MPW-4 exhibited a 10.8% reduction in THR, further substantiating the efficacy of the MCA-modified sodium silicate flame retardant in enhancing the flame-retardant performance of waterborne acrylic coating.

The mass loss rate (MLR) represents the rate at which the sample’s mass decreases during combustion experiments. Figure 6c illustrates that the peak mass loss rate of MPW-4 is 27.2% lower than that of MPW-0, while the peak mass loss rate of MPW-1 is only 15.7% lower than that of MPW-0. This indicates that the poplar veneer treated with flame-retardant coating can effectively suppress combustion, thereby reducing the rate of pyrolysis reaction of the poplar veneer during the combustion test. Therefore, the flame-retardant performance of flame-retardant poplar veneer is superior to that of non-flame-retardant poplar veneer.

The production of smoke is found to increase the risk of asphyxiation, which is often more deadly than the heat from the fire [41]. This is due to the protection of the carbon layer, which results in combustible gas and smoke-forming materials rapidly decreasing in the gas phase during combustion [42,43]. Consequently, accelerating the carbonization process and facilitating the rapid formation of surface carbonization layers represent the most effective means of reducing the transfer rate of external temperature to the interior of flame-retardant wood and reducing the production of combustible volatiles. The total smoke production (TSP) curve comprises two stages: the initial flame combustion process and the subsequent smoldering process [44,45]. Figure 6d,e clearly show that MPW-0 had a significantly higher TSP and TSR than MPW-1 and MPW-4 throughout the entire combustion process. At the initial stage of the combustion test, the total smoke production(TSP) and the total smoke release(TSR) of all samples increased with the extension of time, and then the TSP and TSR curves became gentle. However, in the later smoldering process, the TSP and TSR of all samples began to increase with the extension of time. The TSP of MPW-1 and MPW-4 decreased by 14.7% and 22.4%. The TSR of MPW-1 and MPW-4 decreased by 14.7% and 22.3%. The specific extinction area (SEA) represented the ability to volatize the smoke produced by each unit mass of fuel, which could measure the shading property of smoke [46]. The larger the value was, the larger the smoke produced by the volatile materials [47,48]. As depicted in Figure 6f, during the initial combustion stage, the SEA values of MPW-0, MPW-1, and MPW-4 showed a continuous increasing trend, and their peaks were 382 m^2^·kg^−1^, 357 m^2^·kg^−1^, and 336 m^2^·kg^−1^, respectively, which were 6.5% lower for MPW-1 and 12% lower for MPW-4 compared to MPW-0. Therefore, the addition of sodium silicate nine-hydrate to waterborne acrylic coating can effectively reduce the generation of smoke during the combustion process, while the addition of MCA further effectively reduces the total smoke release. These results indicate that the poplar veneer treated with sodium silicate nine-hydrate and MCA had a significant smoke suppression effect. From Figure 6g–i, it can be seen that there is a difference in the morphology of MPW-4 compared to MPW-0. The residual material after MPW-0 combustion is mainly gray-white powder ash, while MPW-4 char appears black-gray. The cracking of residual carbon after MPW-1 combustion is severe, while the cracking of residual carbon after MPW-4 combustion is better. This indicates that the addition of MCA can promote the formation of a stable, dense, and regular carbon layer structure, effectively suppressing heat transfer during combustion and exhibiting an excellent flame-retardant effect. The flame-retardant effect of the coating is shown in Figure 6j. The flame-retardant effect of sodium silicate is mainly achieved by hydrolyzing into polysilicic acid [49], generating inorganic silicon slag [50], and forming a barrier layer on the surface of wooden materials. MCA plays the role of acting as a gas source in the flame retardant process, which can reduce the surface temperature of the material and dilute O_2_, thus realizing heat insulation and flame retardancy.

### 2.7. The Mechanism of Sodium Silicate/MCA/Waterborne Acrylic Acid Flame-Retardant Coating

**Figure 7:**
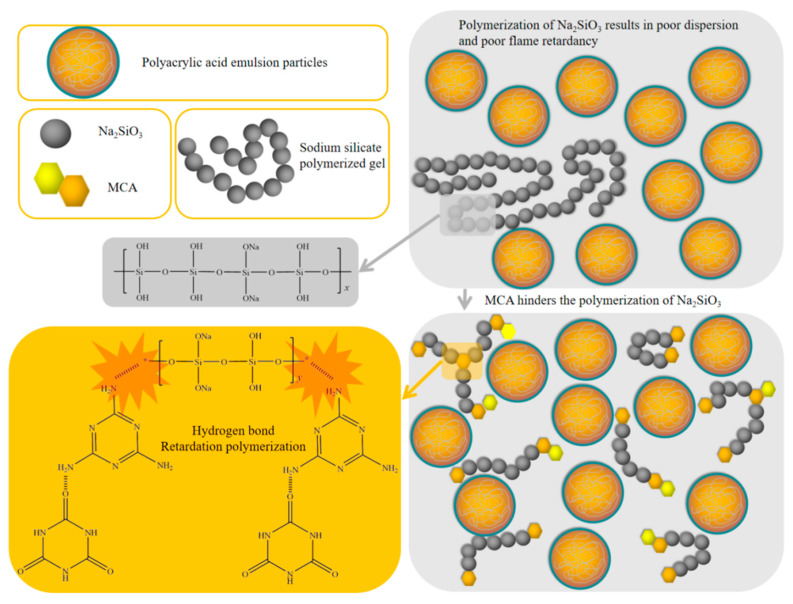
Flame-retardant mechanism of sodium silicate/MCA/waterborne acrylic acid flame-retardant coating.

Figure 7 illustrates the dispersion mechanism of MCA on sodium silicate. Sodium silicate represents an inorganic material that is poorly compatible with organic water-soluble acrylic acid. The agglomeration of sodium silicate within aqueous acrylics affects the acrylic film-forming properties and reduces the flame retardancy of the coating. The literature has demonstrated that reducing, or eliminating, the interface between the organic and inorganic phases in hybrid composites significantly enhances their performance [51,52,53]. Consequently, the incorporation of MCA into sodium silicate impeded the polymerization of sodium silicate through the formation of hydrogen bonds between the amino group of MCA and sodium silicate [54]. The uniform dispersion of sodium silicate in the coating enhanced its flame retardancy. As shown in Figure 6j, by the interaction between MCA and sodium silicate, sodium silicate forms a uniform silica barrier on the surface of wood materials, which effectively provides heat insulation and flame retardancy. In addition, MCA contains a large amount of nitrogen, and during the combustion process, MCA decomposes to produce nitrogen gas. It not only dilutes the surrounding oxygen, but also causes the volume of the coating to expand, forming an expansion layer. This carbon layer effectively inhibits the transfer of heat and volatiles, preventing further damage to the wood substrate. Consequently, the flame-retardant mechanism of the sodium silicate/MCA/waterborne acrylic flame-retardant coating can be described as a typical silicon and nitrogen synergistic expansion flame-retardant mechanism.

## 3. Materials and Methods

### 3.1. Materials

Waterborne acrylic coating (C_3_H_4_O_2_, E0504) was acquired from Shenzhen Jitian Chemical Co., Ltd., Shenzhen, China. Sodium silicate nine-hydrate (AR) was acquired from Sinopharm Chemical Reagent Co., Ltd., Shanghai, China. Melamine cyanurate (MCA, AR) was acquired from Shandong Youso Chemical Technology Co., Ltd., Shandong, China. Deionized water was made in the laboratory. Poplar veneer purchased from Hangzhou Senrui Co., Ltd., Hangzhou, China. Poplar veneers were cut into a size of 300 mm × 300 mm × 2 mm; their moisture content was about 10%.

### 3.2. Preparation of Poplar Veneer

Poplar veneers were sanded to remove the burr, depression, and other defects on its surface so as to make the poplar veneers flatter. Then, the prepared poplar veneers were cut into 65 mm × 65 mm × 2 mm (length × width × thickness) samples.

### 3.3. Preparation of Sodium Silicate/MCA/Waterborne Acrylic Flame Retardant Coating

A quantity of 17.5 g of waterborne acrylic coating was put into the beaker; then, 7.125 g of sodium silicate and 0.375 g of melamine cyanurate were added into the beaker. At that point, the amount of flame retardant added (the percentage of flame-retardant mass to the quality of flame-retardant coating) was 30% and the flame retardant ratio was 5%. The above three samples in the beaker were mixed; then, the beaker was put in a constant temperature oil bath at a temperature of 40 °C and the rotation speed was set at 10 rpm, full mixing and synthesis time was about 20 min. At last, the waterborne acrylic flame-retardant coating whose flame retardant ratio was 5% was prepared. According to the above methods, preparation of the other three waterborne acrylic flame-retardant coatings with flame retardant ratios of 7%, 9%, and 11% was continued. The flame-retardant coatings prepared were named MP-2, MP-3, MP-4, and MP-5. The waterborne acrylic coating without flame retardant was named MP-0, and the group without MCA was named MP-1. After drying, the samples flame-retardant coatings were expressed for the application of MPW-0, MPW-1, MPW-2, MPW-3, MPW-4, and MPW-5. Table 4 shows the experimental sample numbers specifically.

### 3.4. Preparation of Flame-Retardant Poplar Veneers

Twelve pieces of 65 mm × 65 mm poplar veneer sample were coated with waterborne acrylic flame-retardant coatings with different flame retardant ratios (5%, 7%, 9%, and 11%). To reduce errors, each experiment is repeated three times. The coating amount of waterborne acrylic flame-retardant coating was 300 g/m^2^. After brushing, the samples were put into the oven for 8 h, during which the oven was kept at a constant temperature of 60 °C. The samples were removed after drying and stood at room temperature for 48 h. Finally, the poplar veneers after flame-retardant treatment were obtained.

Control group: 17.5 g of waterborne acrylic was weighed; the surfaces of three poplar veneers were evenly coated with the waterborne acrylic. The preparation process is consistent with the preparation process of flame-retardant poplar veneers.

### 3.5. Characterization

Coating viscosity test. The sample of waterborne acrylic flame-retardant coatings with different flame retardant ratios and pure waterborne acrylic acid were poured into a QSG format pipe viscosity meter. The pipe viscosity meter stood in a 25 °C thermostatic water bath for 10 min after the liquid level had risen to the 100 mm scale line. Once the liquid level had reached a stable state, the horizontal handheld viscometer was inverted by 180° to determine the viscosity. Then, the time for the bubble to rise in the viscosity gauge to the top was recorded. Each sample was measured three times, for which the average time was calculated for each measurement with a relative error of 3%.

Paint film hardness test. The QHQ-A portable pencil scratch tester was employed to assess coating samples at room temperature (23 ± 2 °C). Pencils of varying hardness were selected to conduct a pencil scratch test on the coating. In the event that the scratch on the coating of the pencil is less than 3 mm in depth, it is advisable to select a pencil of greater hardness in order to conduct a further pencil scratch test. In the event that the scratch on the coating of the pencil exceeds 3 mm, it is necessary to utilize a softer pencil to repeat the pencil scratch test until no further scratches exceeding 3 mm are observed. The hardness of the paint film is determined by the hardness of the pencil with the hardest scratch, which is 3 mm.

Paint film adhesion test: The test was performed at room temperature (23 ± 2 °C) using the cross-cut tester model QFH-A, purchased from Applied Metrology Ltd., Hangzhou, China. First, the sample was placed on a plate with enough hardness, holding the handle of the knife, so that the sample plane was cut with uniform pressure, both smooth and steadily shaking, and 20–50 mm/s cutting speed with a multi-blade cutting knife. The sample was rotated 90 degrees; then, the above operation was repeated on the cut to form a lattice pattern. Finally, the diagonal on both sides of the lattice pattern was swept with a soft brush to gently brush backward 5 times, and brush forward 5 times. The film adhesion grade was determined according to the percentage of the film number, bound using tape.

Ignition testing: Experiments were designed according to the national standard GB 8624-2012. Measure oxygen index according to standard ASTM D2836 (JF-3 oxygen index meter). Each group of samples was repeated 10 times. X-ray diffraction (XRD) was carried out on an XRD-D2 produced by the German Brooke Company, Saarbrücken, Germany. The scanning range was 10°–80° (2θ) and the scanning speed was 6°/min. Raman was tested and analyzed by theLabRam HR Evolution, the laser wavelength was selected to be 532 nm, and the test range was 700–1500 cm^−1^. Particle size tests were performed in a Welas-type particle size spectrometer from Palas, Karlsruhe, Germany. The morphology and dispersion of sodium silicate/MCA/waterborne acrylic flame-retardant coating were observed via the SU8010-type cold field emission SEM produced by Hitachi, Tokyo, Japan. The sample elements were investigated in combination with SEM to detect the relative content of the sample. TG curves were obtained by using the HTG-2 thermogravimetric analyzer with a temperature range of 25–1000 °C and a heating rate of 30 °C/min. Finally, the flame retardancy of non-flame-retarded poplar veneer and flame-retarded poplar veneer (100 mm × 100 mm × 4 mm) was tested using a cone calorimeter (CONE) manufactured by Nechi Instruments GmbH, Bavaria, Germany. The power of the thermal radiation was set at 50 kW/m^2^ to bring the experimental temperature close to the fire temperature. All experiments were repeated three times.

## 4. Conclusions

In this study, a novel sodium silicate/MCA/waterborne acrylic flame-retardant coating was prepared with MCA-modified sodium silicate as a flame retardant in order to improve the dispersion of sodium silicate in acrylic acid and to act as a gas source co-efficient flame retardant. The flame-retardant treatment was applied to poplar veneer to prepare flame-retardant poplar veneer. The relevant properties and mechanisms of the flame-retardant coating and the flame-retardant poplar wood veneer were investigated. Compared with the sodium silicate/MCA/waterborne acrylic flame-retardant coatings, the former all showed milky white color, no crystallization and agglomeration phenomenon, as well as uniform dispersion. When the ratio of the flame retardant in the flame-retardant coating was 9% (MPW-4), the viscosity of the flame-retardant coating was comparable to that no flame retardant, the hardness of the paint film reached HB, and the adhesion of the paint film reached 4B. The flame retardant did not degrade the physical properties of the coating. According to the analysis of XRD, particle size, SEM, and EDX, it was found that the distribution of Si on the surface of flame-retardant poplar veneer was more uniform, and the phenomenon of agglomeration did not occur; the Si contained in the flame-retardant coatings were reduced, and the Na and O increased. The particle size of sodium silicate/MCA/waterborne acrylic flame-retardant coating decreased, the Si peak was no longer sharp, and the degree of crystallinity decreased significantly. Thus, it was shown that the addition of MCA can improve the agglomeration phenomenon of sodium silicate and improve the dispersion of sodium silicate in acrylic acid. A comparison was made between the MPW-0 flame retardant-treated poplar veneer and the MPW-4 fire retardant-treated poplar veneer. The residual charcoal layer of the MPW-4 veneer was found to be denser after burning, with a substantial increase in thickness. Additionally, the mass fraction of residue increased by 14.4%. The ignition time was increased by 122.7%, the limiting oxygen index value was increased by 43.0%, the heat release rate (HRR) was decreased by 19.9%, the total heat release (THR) was decreased by 10.8%, and the mass loss rate was decreased by 27.2%. Consequently, the poplar veneer treated with a sodium silicate/MCA/waterborne acrylic flame-retardant coating exhibited excellent flame-retardant properties. The flame retardancy of aqueous acrylic-based sodium silicate/MCA flame-retardant coatings is a typical silicon–nitrogen synergistic expansion-type flame-retardant mechanism. In this paper, a novel method was presented to improve the dispersion of inorganic flame retardants, which markedly enhanced the dispersion and homogeneity of inorganic flame retardants in organic polymers, and significantly enhanced the flame-retardant properties of the coatings. Poplar veneer treated with a sodium silicate/MCA/waterborne acrylic flame-retardant coating exhibited excellent flame-retardant properties. This study provides a new method to improve the effective and homogeneous dispersion performance of inorganic flame retardants in organic coatings, which is of great significance to promoting the vigorous development and application of new flame-retardant coatings.

## Figures and Tables

**Table 1:** Paint film hardness and adhesion of untreated poplar veneer and flame retardant-treated poplar veneer.

Name	MPW-0	MPW-1	MPW-2	MPW-3	MPW-4	MPW-5
Paint film hardness	HB	HB	H	HB	HB	HB
Paint film adhesion	4B	4B	4B	4B	4B	4B

**Table 2:** Limiting oxygen index of untreated poplar veneer versus fire retardant-treated poplar veneer.

Name	Poplar Veneer	MPW-0	MPW-1	MPW-2	MPW-3	MPW-4	MPW-5
LOI (%)	20.7	20.0	27.0	27.9	28.0	28.6	26.8

**Table 3:** Mean parameter values of the samples.

Name	Mean						
	Time to Ignition (s)	HRR (kW/m^2^)	THR (MJ/m^2^)	MLR (g/s)	TSP (m^2^)	TSR (m^2^/m^2^)	SEA (m^2^/kg)
MPW-0	19 ± 1	634.12 ± 3.98	24.48 ± 0.65	0.49 ± 0.04	1.52 ± 0.07	143.05 ± 13.64	382.46 ± 10.53
MPW-1	24 ± 0	624.77 ± 4.14	24.38 ± 0.49	0.41 ± 0.03	1.12 ± 0.02	120.87 ± 11.83	357.29 ± 16.23
MPW-4	28 ± 1	508.82 ± 9.11	21.46 ± 0.77	0.36 ± 0.03	1.09 ± 0.03	109.02 ± 4.4	336.41 ± 10.79

**Table 4:** Experimental samples.

Sample Number	Sodium Silicate/MCA/Waterborne Acrylic Flame-Retardant Coating		Flame-Retardant Poplar Veneer
	MCA/(MCA+ Sodium Silicate)	Sodium Silicate/(MCA+ Sodium Silicate)	
MP-0	0	0	-
MP-1	0	100%	-
MP-2	5%	95%	-
MP-3	7%	92%	-
MP-4	9%	91%	-
MP-5	11%	89%	-
MPW-0	-	-	Poplar veneer treated with MP-0
MPW-1	-	-	Poplar veneer treated with MP-1
MPW-2	-	-	Poplar veneer treated with MP-2
MPW-3	-	-	Poplar veneer treated with MP-3
MPW-4	-	-	Poplar veneer treated with MP-4
MPW-5	-	-	Poplar veneer treated with MP-5

## Data Availability

No new data were created or analyzed in this study. Data sharing is not applicable to this article.
